# Potential value of pre-planned imaging of bone disease in multiple myeloma

**DOI:** 10.1038/s41408-023-00880-0

**Published:** 2023-07-07

**Authors:** Michael T. Gundesen, Jon Thor Asmussen, Fredrik Schjesvold, Annette Juul Vangsted, Carsten Helleberg, Einar Haukås, Trine Silkjær, Elena Manuela Teodorescu, Bo Amdi Jensen, Tobias S. Slørdahl, Hareth Nahi, Anders Waage, Niels Abildgaard, Thomas Lund, Nordic Myeloma Study Group

**Affiliations:** 1 Department of Hematology, Odense University Hospital, Odense, Denmark; 2 Department of Clinical Research, University of Southern Denmark, Odense, Denmark; 3 Department of Radiology, Odense University Hospital, Odense, Denmark; 4 Oslo Myeloma Center, Department of Hematology, Oslo University Hospital, Oslo, Norway; 5 K.G. Jebsen Centre for B-Cell Malignancies, University of Oslo, Oslo, Norway; 6 Department of Hematology, Rigshospitalet, Copenhagen, Denmark; 7 Department of Blood and cancer diseases, Stavanger University Hospital, Stavanger, Norway; 8 Department of Hematology, Aarhus University Hospital, Aarhus, Denmark; 9 Department of Hematology Aalborg University Hospital, Aalborg, Denmark; 10 Department of Hematology, Zealand University Hospital, Roskilde, Denmark; 11 Department of Hematology, St. Olavs Hospital - Trondheim University Hospital, and Norwegian University of Science and Technology (NTNU), Trondheim, Norway; 12 Karolinska Institutet, Solna, Sweden

**Keywords:** Medical imaging, Cancer imaging

Dear Editor,

International consensus for monitoring bone disease in multiple myeloma (MM) by imaging is to perform these at disease progression or clinical suspicion [1].

Still MM is a heterogeneous disease where progression of lytic bone disease can be observed in patients in biochemical remission without symptoms. In one study progression of lytic bone disease was seen before biochemical progression in 15% of patients [2].

Studies exploring pre-planned sequential imaging are limited and have not revealed substantial clinical value, possibly because of the low sensitivity of conventional radiography [3–5]. WBLDCT (Whole Body Low Dose CT) has demonstrated superiority compared to conventional radiography in detecting bone lesions [4], and therefore WBLDCT has replaced conventional radiography as the gold standard [1].

Progressive osteolytic disease traditionally defines clinical progression and need for treatment of MM [6]. More findings of progressive bone disease with standard use of WBLDCT instead of conventional X-ray could allow earlier treatment and challenge the current recommendations. This investigation of preplanned imaging is a sub-study of the Magnolia study in MM (EudraCT number 2014-002494-12).

Magnolia investigates the effect of giving zoledronic acid (ZOL) for 2 vs 4 years. We prospectively monitored newly diagnosed patients with symptomatic MM for up to 4 years after diagnosis. An amendment allowed patients treated with ZOL for 2 years to be included at 2-year-randomization. Study centers in Denmark and Norway did monthly outpatient visits, disease evaluations and blood biobanking. Further, quality of life (QoL) questionnaires, EORTC QLQ CTC30, were conducted every 6 months the first 2 years and every 3 months from year 2-4 and WBLDCT was performed according to guidelines [1]. In addition, pre-planned WBLDCT was conducted at 0, 12, 24, 30, 36, 42, 48 months after diagnosis. The reason for increased frequency of QoL measurements and WBLCT after randomization at 24 months was to strengthen comparison of continued ZOL and stopped treatment. All imaging was evaluated compared to latest imaging and imaging taken at inclusion by a local radiologist. Progressive bone disease (PBD) was defined: ”≥25% increase in size of existing osteolytic lesions or new osteolytic lesions (both at least 10 mm increase/diameter), fractures, spontaneous fractures or lesions needing irradiation therapy or surgery”. Detailed description of methods are shown in Supplementary material. The trial was performed in accordance with the Declaration of Helsinki and approved by Regional Committees on Health Research Ethics for Southern Denmark (EPN S-20140138) and Norway (EPN REK 2015/626).

## Results

**Fig. 1:**
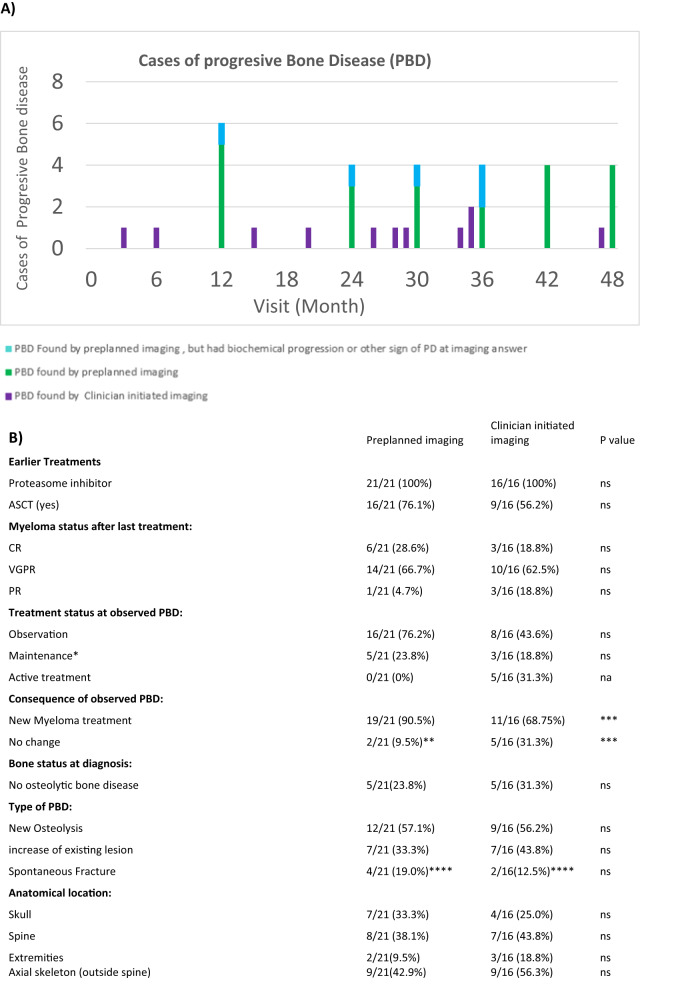
Findings of progressive bone disease over time in newly diagnosed multiple myeloma. **a** Overview of findings of progressive bone disease (PBD) ”PBD clinician-initiated imaging” shows cases found due to imaging performed on clinical suspicion of progressive bone disease and cases with biochemical progression or CRAB, ”PBD: preplanned imaging” are cases of progressive bone disease found due to preplanned imaging. 70.3 % of all PBD cases in our study were found by preplanned imaging investigation with whole body low dose-CT. Preplanned imaging investigations were performed at months 12 (*n* = 89), 24 (*n* = 79), 30 (*n* = 181), 36 (*n* = 164) 42 (*n* = 126) and 48 (*n* = 93). **b** Description of findings since last imaging, earlier treatments, type and placement of lesions and results of last treatments for the cases of progressive bone disease seen in **a**. ”New osteolysis” was defined as a new osteolytic lesion of at least 10 mm and “increase of existing lesion” was defined as increase in size of existing osteolytic lesion of at least 25% and at least 10 mm. * Reimbursement for maintenance with lenalidomide was not approved until autumn 2019. **One patient was reevaluated with MR in which lesions were less than on WBLDCT. *** The 5 cases where treatment was not initiated in the found by clinician group was due to already being. in active treatment, which was deemed effective. **** Three cases in PBD found by preplanned imaging had spontaneous fracture only (month 12), One case in the PBD found by clinician initiated imaging (month 26).

In total, 840 WBLDCTs were performed in 267 patients. Median follow-up was 22.3 months. Patient characteristics are presented in Table 1. A total of 37 cases of PBD were detected. (Fig. 1a).

**Table 1:** Patient characteristics at diagnosis.

	All		Cases of PBD		
Age mean (range)	65.1 years	(42–83)	65.0 years	(48–82)	
	*n*	%	*n*	%	
Gender (Male)	163	61.0	25	67.6	ns
Myeloma:					
IgG	164	67.7	23	62.2	ns
IgA	48	14.6	8	21.6	ns
Light chain	55	17.7	6	16.2	ns
ISS at diagnosis					
I	119	44.5	12	32.4	ns
II	97	36.3	19	51.4	ns
III	46	17.7	6	16.2	ns
Missing	5	1.9	0	0.0	ns
R-ISS at diagnosis					
I	69	25.8	5	18.5	ns
II	109	40.8	19	51.4	ns
III	31	11.6	5	18.5	ns
Missing	58	21.7	8	17.0	ns
Performance status (inclusion)					
0	157	58.8	14	37.8	*P* < 0.01
1	82	30.7	12	32.4	ns
2	15	5.6	5	13.5	ns
3	7	2.6	6	16.2	*P* < 0.01
4	1	0.4	0	0.0	ns
Missing	5	1.9	0	0.0	ns
Cytogenetics					
t(4;14)	13	4.9	1	2.7	ns
t(14;16)	5	1.9	1	2.7	ns
del17p	12	4.5	2	5.4	ns
none of above	121	45.3	20	54.1	ns
missing	91	34.1	13	35.1	ns

Patient characteristics. Distribution of gender, age, myeloma type, ISS, R-ISS and cytogenetics, diagnosis and performance status at diagnosis of patients included in study in all patients and in patients where progressive bone disease was found.

Eleven cases of PBD were found in the study period by WBLDCT done by clinical indication, hereof 1 case of vertebral collapse. Pre-planned imaging added 26 PBD cases. Of these 26 cases, however, four had biochemical progression and 1 had significant anemia. Using current imaging recommendations these 5 cases should have been found by clinician initiated imaging [1]. Reclassifying these 5 patients, still 21 cases of PBD were identified by pre-planned imaging, hereof 3 cases of spontaneous fracture (Fig. 1).

QoL questionnaire data (filled before visit where suspicion was raised), in participants who later were identified with PBD on WBLDCT ordered by clinicians suspicion, reported clinically relevant worse overall health, physical function, role functioning, social functioning, emotional functioning, and more pain, fatigue, insomnia and general disease symptoms compared to patients that were found to have PBD on pre-planned imaging. No differences were observed for the domains cognitive function, future perspective and body image. The QoL results can be found in Supplementary material.

The findings of PBD with pre-planned imaging did lead to a change of treatment in 19 of 21 cases. One case was further evaluated by Magnetic Resonance imaging and found to be less evident progression than indicated by WBLDCT and another case being a spontaneous fracture which was deemed not to require additional treatment. For PBD found on clinician suspicion 11/16 cases lead to new treatment whereas the remaining 5 already had started new treatment before imaging based on other indication. Most (28/37) found with PBD had achieved less than CR during last treatment while 9 had achieved CR. (Fig. 1b)

Execution of clinically indicated imaging was not delayed due to upcoming preplanned imaging, as the rate of investigations per month per patient was 0.026 1 and 2 months before pre-planned imaging compared to 0.020 3 to 4 months prior to pre-planned imaging. The difference was insignificant and a clear indication that clinicians did not delay well indicated imaging because of imminent pre-planned imaging.

The positive rate of identified PBD by pre-planned WBLDCT was 3.6% in total, 6.1% when performed annually and 2.8% if performed every 6 months. The positive rate of identified PBD by clinically initiated WBLDCT was significantly higher at 9.9% (*p* = 0.04).

## Discussion

Progression of existing lesions or emergence of new lesions are criteria for clinical progression and calls for re-initiation of treatment or a shift of current treatment [7]. In our study, 21 cases of PBD were found by pre-planned imaging, with 16 of these leading to change of treatment.

In a study by Gavriatopoulou et al. 100 patients with SMM (smoldering myeloma) were prospectively followed for up to 5 years with pre-planned WBLDCT conducted yearly. Of 31 patients with myeloma progression, 10 patients were only identified due to bone lesions found on protocol-scheduled WBLDCT [8]. Although SMM is different from MM, in both settings a substantial number of patients with PBD are only identified with preplanned imaging. A former study using conventional radiography did not find clinical value of serial investigations [3]. This difference is possibly caused by higher sensitivity of WBLDCT [4, 5, 9]. However, it should be noted that a smaller study similarly found PBD in some patients without additional findings [2].

Early detection of PBD are intuitively important offering initiation of effective treatment in a timely manner. This is supported by our QoL data. Patients where detection was delayed until clinically suspected progression reported more pain, fatigue and disease symptoms. Early identified PBD is not only relevant for initiating anti-myeloma treatment, as the AZABACHE study demonstrated that early re-initiation of ZOL can reduce incidence of further PBD [10].

A drawback to consider is that pre-planned serial imaging would come with additional costs: economic, logistic and increased radiation burden. WBLDCT protocols are fast and comfortable for patients, and significantly reduce radiation exposure (<5 mSv) compared to standard dose CT [11, 12]. The additional radiation burden could be eliminated using magnetic resonance imaging, however this will increase logistics, the cost of time and patient comfort. Overall, according to our data the number of investigations performed to identify cases with PBD would increase.

In our study PBD was found in 3.6% of pre-planned imaging vs in 9.9% of clinician initiated imaging. A 2015 study by Zamagni et al. with serial PET-CT found decreased risk of exclusive bone progression in patients achieving complete metabolic response [13]. Most of our patients with PBD (28/37) had achieved less than CR during last treatment. Our study cannot show if achieving CR would reduce risk of PBD. ISS/R-ISS or cytogenetics was not prognostic, whereas WHO PS at diagnosis was different in the group who experienced PBD in the study (Table 1).

As shown by Raje et al. [14] new bone related events are more common in the first 3 months of treatment with ZOL or denosumab, before full effect of treatment have been achieved. This does not necessarily represent treatment failure. We identified five cases of PBD at pre-planned WBLDCT at 12 months, hereof three cases of spontaneous fracture. These cases may have been such very early events that will normally not be interpreted as progression if the treatment causes a biochemical response. As most patients with PBD found by pre-planned imaging (20/21) had achieved CR/VGPR, we considered if some lesions could have occurred during last MM treatment. However, except for the five cases mentioned, all had at least one imaging performed without new findings since end of last treatment. Still care should be taken not to over-interpret and thereby over-treat patients. In doubtful cases supplementary imaging such as MRI or PET/CT will be helpful.

Another approach for monitoring bone disease in MM could be the use of serum or urine markers of bone resorption and bone formation. A study from 2016 investigated if the interval between zoledronic acid infusion could be directed by measuring urine NTX and found that this approach was associated with a low number of skeletal events [15]. However, more data is needed before this approach can be recommended for daily practice.

In conclusion, adding regular pre-planned bone imaging to the current guidelines more than doubled the number of patients with identified PBD. Patients with detected PBD after clinical suspicion reported more pain and reduced QoL. Thus, using regular bone imaging, patients with PBD could be identified at an earlier time point enabling adjusted treatment and efforts before severe bone damage occur. We encourage more studies to be done in this area. If our findings are confirmed it will potentially change the recommendation on how to monitor myeloma bone disease in future guidelines.

### Supplementary information

Supplemental material

## Data Availability

The datasets generated during and/or analyzed during the current study are not publicly available due to privacy or ethical restrictions but are available from the corresponding author on reasonable request.
