# Supplementary material for: Potential value of pre-planned imaging of bone disease in multiple myeloma
Source: Blood Cancer J. 2023 Jul 7;13(1):105. doi: 10.1038/s41408-023-00880-0 (PMC10328913; doi:10.1038/s41408-023-00880-0)
Supplement: Supplementary file 1 — Supplemental material [file 41408_2023_880_MOESM1_ESM.docx]

**Methods:**

This study is a sub-study of the WBLDCT data generated in the prospective Magnolia Study conducted by the Nordic Myeloma Study Group (EudraCT number 2014-002494-12). The Magnolia Study is an investigator-initiated, randomized, multinational phase 3 clinical trial exploring whether four years zoledronic acid treatment is superior to only two years of treatment. Here we analyzed data from a total of 267 patients included in the Magnolia study, 156 patients with newly diagnosed treatment-demanding MM and 111 patients with MM that were included two years after initial diagnosis. The 111 patients were included after an amendment allowing patients to be included, if they after MM diagnosis had received two years of treatment with zoledronic acid. These patients proceeded directly to randomization, to stop or to continue zoledronic acid for two more years. Thus, all were treated with zoledronic acid for two years before 1:1 randomization to two more years of treatment or observation. All patients were followed until four years after diagnosis or until they left the protocol. For ethical reasons, participants were not allowed to proceed to randomization after two years of zoledronic acid treatment, if they had demonstrated progressive bone disease within three months prior to planned randomization. Data were collected from March 2015 to December 2021.

During the entire study period, all patients had blood samples taken monthly including, hemoglobin, leucocytes including differential count, thrombocytes, ionized calcium, creatinine, immunoglobulin A, G and M, M-component and serum free light chains kappa and lambda. Participants were evaluated monthly at a department of hematology and in case of clinical suspicion of treatment-demanding relapse or clinical suspicion of PBD, new imaging was performed. The protocol did not demand new imaging at biochemical progression, as the protocol was initiated before this was introduced in the 2019 IMWG guidelines(1). Progressive disease was defined according to the 2016 IMWG(2)criteria.

Pre-planned WBLDCT was conducted in all patients 0, 12, 24, 30, 36, 42, and 48 months after start of treatment and in patients included at diagnosis, and 24, 30, 36, 42, and 48 months after start of treatment in patients included after 24 months. Only, pre-planned imaging was abandoned if an unscheduled WBLDCT was done within 3 months; this to reduce unnecessary radiation exposure. The reason for conducting an unscheduled imaging was registered to avoid misclassification by temporal coincidence. Patients on zoledronic acid left the protocol in case of bisphosphonate-induced osteonecrosis of the jaw, and patients in observation left the protocol in case of progressive bone disease. In both situations data until leaving protocol was included in analysis.

Imaging:

All patients were examined with WBLDCT systems used at their local hospital. Test images were approved by the leading project radiologist before a site could be initiated. Participants were placed in a supine position with arms raised above their shoulders, alongside the head, when shoulder motion-range allowed it. The scanned field spanned from vertex/elbows to the knees. All imaging was evaluated by a local radiologist for PBD following criteria pre-defined in the protocol. PBD was defined as ”≥ 25% increase in size of existing osteolytic lesions (a total increase of at least 10 mm is required in the longest dimension) or new osteolytic lesions (at least 10 mm in the longest dimension) or fractures, spontaneous fractures, or lesions needing irradiation therapy or surgery”. All imaging was evaluated compared to latest imaging and imaging taken at inclusion.

QoL was evaluated at inclusion and every 6 months for the first 2 years after diagnosis and hereafter every 3 months. Patients answered the EORTC QLQ-C30(3), version 3.0 and the EORTC QLQ-MY20, version 3.0(4) in their local language (Danish/Norwegian). Data scoring was performed as recommended in the EORTC scoring manual(5). Clinical value of changes was evaluated as trivial (no clinical significance, small or moderate) according to guidelines from Cocks et al(6) for EORTC QLQ-C30 and Sully et al(7) for EORTC QLQ-MY20.

QoL questionnaires were answered on the same visit date that pre-planned WBLDCT was ordered to ensure that no new information of bone status was known by the participants until after questionnaires had been answered. In cases of progressive bone disease found by imaging initiated by clinicians, the latest QoL questionnaire answers from before the WBLDCT investigation were used to avoid knowledge of imaging results affecting answers.

Statistics were calculated using Stata BE 17. Difference in proportions was evaluated using Z-test for difference in proportions. Difference in means was evaluated using an unpaired t-test between groups and a paired t-test for paired data with changes over time. Chi-squared test was used for comparison of groups.

| **Quality of life scores at latest questionnaire prior to PBD being discovered** | **By clinician (n = 10)** | **By screening (n = 23)** | **Difference** | **Clinical significance** |
| --- | --- | --- | --- | --- |
| **Overall (0 -100 higher score better)** | | |  |  |
| Global Health | 55.56 | 65 | -9.44 | small |
| **Functional Scales (0 -100 higher score better)** | | | |  |
| Physical functioning | 67.4 | 73.33 | -5.93 | small |
| Role functioning | 48.15 | 64.16 | -16.01 | medium |
| Emotional functioning | 79.63 | 85 | -5.37 | small |
| Cognitive functioning | 85.18 | 84.16 | 1.02 | trivial |
| Social Functioning | 70.37 | 83.33 | -12.96 | medium |
| *Future perspective* | 69.13 | 67.77 | 1.36 | trivial |
| *Body image* | 77.77 | 76.66 | 1.11 | trivial |
|  |  |  |  |  |
| **Symptom scales/items (0 - 100 Lower score better)** | | | | |
| Fatigue | 40.74 | 31.67 | 9.07 | small |
| Nausea and vomiting | 7.4 | 2.5 | 4.9 | trivial |
| Pain | 33.33 | 23.33 | 10 | small |
| Dyspnea | 18.52 | 11.67 | 6.85 | small |
| Insomnia | 22.22 | 8.33 | 13.89 | medium |
| Appetite loss | 22.22 | 15 | 7.22 | small |
| Constipation | 18.52 | 10 | 8.52 | small |
| Diarrhea | 29.62 | 11.66 | 17.96 | medium |
| Financial difficulties | 11.11 | 0 | 11.11 | medium |
| Disease symptoms | 27.16 | 18.33 | 8.83 | small |
| Side effects of treatment | 14.93 | 12.54 | 2.39 | trivial |

Table 1: Quality of life in patients with progressive osteolytic disease found by clinician compared to cases found by preplanned monitoring. Scores showing better QoL for preplanned imaging marked with green. No clinical difference (trivial) non-colored. There were no areas with better QoL in cases found by clinicians.

A total of 50 questions were scored by patients regarding 19 areas of quality of life in EORTC QLQ-C30(3), version 3.0 and the EORTC QLQ-MY20, version 3.0(4) questionnaires. Scores from 0 to 100 are calculated according to scoring manual(5). For overall and functional scales, 100 is perfect health, while for symptom scores, 100 is worst possible. Clinical difference is evaluated according to recommendations for the questionnaires(6, 7). Patients with progressive bone disease found by pre-planned monitoring have lower symptoms and better function in almost all areas.

1. [Hillengass](https://pubmed.ncbi.nlm.nih.gov/?term=Hillengass+J&cauthor_id=31162104) J , [Usmani](https://pubmed.ncbi.nlm.nih.gov/?term=Usmani+S&cauthor_id=31162104) S , [Rajkumar](https://pubmed.ncbi.nlm.nih.gov/?term=Rajkumar+SV&cauthor_id=31162104) SV , [Durie](https://pubmed.ncbi.nlm.nih.gov/?term=Durie+BGM&cauthor_id=31162104) BGM , [Mateos](https://pubmed.ncbi.nlm.nih.gov/?term=Mateos+MV&cauthor_id=31162104) MV , [Lonial](https://pubmed.ncbi.nlm.nih.gov/?term=Lonial+S&cauthor_id=31162104) S et. al , *International myeloma working group consensus recommendations on imaging in monoclonal plasma cell disorders.* Lancet Oncol, 2019. **20**: p. e302–12.

2. [Kumar](https://pubmed.ncbi.nlm.nih.gov/?term=Kumar+S&cauthor_id=27511158) S , [Paiva](https://pubmed.ncbi.nlm.nih.gov/?term=Paiva+B&cauthor_id=27511158) B , [Anderson](https://pubmed.ncbi.nlm.nih.gov/?term=Anderson+KC&cauthor_id=27511158) KC , [Durie](https://pubmed.ncbi.nlm.nih.gov/?term=Durie+B&cauthor_id=27511158) B, [Landgren](https://pubmed.ncbi.nlm.nih.gov/?term=Landgren+O&cauthor_id=27511158) O , [Moreau](https://pubmed.ncbi.nlm.nih.gov/?term=Moreau+P&cauthor_id=27511158) P  et. al. *International Myeloma Working Group consensus criteria for response and minimal residual disease in multiple myeloma.* Lancet Oncology, 2016. **17**. p. 328-46

3. [N K Aaronson](https://pubmed.ncbi.nlm.nih.gov/?term=Aaronson+NK&cauthor_id=8433390), [S Ahmedzai](https://pubmed.ncbi.nlm.nih.gov/?term=Ahmedzai+S&cauthor_id=8433390), [B Bergman](https://pubmed.ncbi.nlm.nih.gov/?term=Bergman+B&cauthor_id=8433390), [M Bullinger](https://pubmed.ncbi.nlm.nih.gov/?term=Bullinger+M&cauthor_id=8433390), [A Cull](https://pubmed.ncbi.nlm.nih.gov/?term=Cull+A&cauthor_id=8433390), [N J Duez](https://pubmed.ncbi.nlm.nih.gov/?term=Duez+NJ&cauthor_id=8433390) et. al.. The European Organization for Research and Treatment of Cancer QLQ-C30: A Quality-of-Life Instrument for Use in International Clinical Trials in Oncology. . J Natl Cancer Inst. 1993;85:365–76.

4. [K Cocks](https://pubmed.ncbi.nlm.nih.gov/?term=Cocks+K&cauthor_id=17574838) , [D Cohen](https://pubmed.ncbi.nlm.nih.gov/?term=Cohen+D&cauthor_id=17574838), [F Wisløff](https://pubmed.ncbi.nlm.nih.gov/?term=Wisl%C3%B8ff+F&cauthor_id=17574838), [O Sezer](https://pubmed.ncbi.nlm.nih.gov/?term=Sezer+O&cauthor_id=17574838), [S Lee](https://pubmed.ncbi.nlm.nih.gov/?term=Lee+S&cauthor_id=17574838), [E Hippe](https://pubmed.ncbi.nlm.nih.gov/?term=Hippe+E&cauthor_id=17574838) et al. An international field study of the reliability and validity of a disease-specific questionnaire module (the QLQ-MY20) in assessing the quality of life of patients with multiple myeloma. European Journal of Cancer. 2007;43(11):1670–8.

5. [Fayers](https://www.researchgate.net/scientific-contributions/Peter-Fayers-39835822?_sg%5B0%5D=xdKEo77tLZpdgzn5Lc7reDb4uMdNI3i_--kn6CKYqncV06vvLBQ_UT2kDn1h5fPclbOxLFI.QfyAjW_xoeHs6HmdqtboTpHfaqDyAXjFXCQWL0k37pKiX1xYzdT2G1QrzGG_z7UfNzknnd2DJjPjwZ9G-vJ-4A&_sg%5B1%5D=itQwp5DFhT8Jp1BsTbiWi2jxTeJzqCGNusFdD5UNtxrllczSxX6qIAH9lg3RJijTthhqDQA.H2pTvp1RK02tfZmsOzO2vV0AsiwhQ5iHvnA5JHJOKj-EDWz_IHOyjn5Y_v1XzMOYBaBr8pyzh5VCSI_zcecbLQ) PM, Aaronson N [Bjordal](https://www.researchgate.net/profile/Kristin-Bjordal?_sg%5B0%5D=xdKEo77tLZpdgzn5Lc7reDb4uMdNI3i_--kn6CKYqncV06vvLBQ_UT2kDn1h5fPclbOxLFI.QfyAjW_xoeHs6HmdqtboTpHfaqDyAXjFXCQWL0k37pKiX1xYzdT2G1QrzGG_z7UfNzknnd2DJjPjwZ9G-vJ-4A&_sg%5B1%5D=itQwp5DFhT8Jp1BsTbiWi2jxTeJzqCGNusFdD5UNtxrllczSxX6qIAH9lg3RJijTthhqDQA.H2pTvp1RK02tfZmsOzO2vV0AsiwhQ5iHvnA5JHJOKj-EDWz_IHOyjn5Y_v1XzMOYBaBr8pyzh5VCSI_zcecbLQ) K

EORTC QLQ-C30 Scoring Manual the EORTC QLQ-C30 Introduction. EORTC QLQ-C30. Scoring Man 2001;30:1-67.

6. [K Cocks](https://pubmed.ncbi.nlm.nih.gov/?term=Cocks+K&cauthor_id=22418017) , [M T King](https://pubmed.ncbi.nlm.nih.gov/?term=King+MT&cauthor_id=22418017), [G Velikova](https://pubmed.ncbi.nlm.nih.gov/?term=Velikova+G&cauthor_id=22418017), [G de Castro Jr](https://pubmed.ncbi.nlm.nih.gov/?term=de+Castro+G+Jr&cauthor_id=22418017), [M Martyn St-James](https://pubmed.ncbi.nlm.nih.gov/?term=Martyn+St-James+M&cauthor_id=22418017) et al . Evidence-based guidelines for interpreting change scores for the European Organisation for the Research and Treatment of Cancer Quality of Life Questionnaire Core 30. J Eur J Cancer. 2012;48:1713–21.

7. [Sully](https://pubmed.ncbi.nlm.nih.gov/?term=Sully+K&cauthor_id=31444815) K , [Trigg](https://pubmed.ncbi.nlm.nih.gov/?term=Trigg+A&cauthor_id=31444815) A , [Bonner](https://pubmed.ncbi.nlm.nih.gov/?term=Bonner+N&cauthor_id=31444815) N , [Moreno-Koehler](https://pubmed.ncbi.nlm.nih.gov/?term=Moreno-Koehler+A&cauthor_id=31444815) A, [Trennery](https://pubmed.ncbi.nlm.nih.gov/?term=Trennery+C&cauthor_id=31444815) C, [Shah](https://pubmed.ncbi.nlm.nih.gov/?term=Shah+N&cauthor_id=31444815) N et al.  . Estimation of minimally important differences and responder definitions for EORTC QLQ‐MY20 scores in multiple myeloma patients. Eur J Haematol 2019;103:500–9.
